# Biophotonic sensor for swift detection of malignant brain tissues by using nanocomposite YBa_2_Cu_3_O_7_/dielectric material as a 1D defective photonic crystal

**DOI:** 10.1038/s41598-023-34601-1

**Published:** 2023-05-19

**Authors:** C. Malek, Suhad Ali Osman Abdallah, S. K. Awasthi, M. A. Ismail, W. Sabra, Arafa H. Aly

**Affiliations:** 1 TH-PPM Group, Physics Department, Faculty of Sciences, Beni-Suef University, Beni Suef, 62514 Egypt; 2 Applied College, Khamis Mushait, King Khalid University, Abha, 62529 Kingdom of Saudi Arabia; 3 Department of Physics and Material Science and Engineering, Jaypee Institute of Information Technology, Noida, 201304 India; 4 Faculty of Technology and Education, Beni-Suef University, Beni Suef, 62521 Egypt; 5 University College in Al Arda, Jazan University, Jazan, 82817 Kingdom of Saudi Arabia

**Keywords:** Materials science, Optics and photonics, Physics

## Abstract

In the present research work we have theoretically examined the biosensing capabilities of proposed one dimensional defective photonic crystal for swift detection of malignant brain tissues. The transfer matrix formulation and MATLAB computational tool have been used to examine the transmission properties of proposed structure. The identical buffer layers of nanocomposite superconducting material have been used either side of cavity region to enhance the interaction between incident light and different brain tissue samples poured into the cavity region. All the investigations have been carried out under normal incidence to suppress the experimental liabilities involved. We have investigated the biosensing performance of the proposed design by changing the values of two internal parameters (1) the cavity layer thickness (*d*_4_) and (2) volume fraction (*η*) of nanocomposite buffer layers one by one to get the optimum biosensing performance from the structure. It has been found that the sensitivity of the proposed design becomes 1.42607 μm/RIU when the cavity region of thickness 15dd is loaded with lymphoma brain tissue. This value of sensitivity can be further increased to 2.66136 μm/RIU with *η* = 0.8. The findings of this work are very beneficial for designing of various bio-sensing structures composed of nanocomposite materials of diversified biomedical applications.

## Introduction

The unusual growth of tissues inside brain or middle of spine may result development of brain tumors. Such tumors may affect the proper functioning of human brain. These brain tumors are grouped into two basic categories as primary and metastatic. Primary brain tumors are developed either from tissues of brain or their immediate surroundings. Primary brain tumors are further classified into glial or non glail and bening or malignant. On the other hand metastatic brain tumors may develop elsewhere in the human body and may infiltrate into brain through human bloodstream. The benign is least aggressive class of tumors. They may exist inside cells or adjoining regions of brain. Such tumors are generally noncancerous and are not responsible for spreading of malignancy into the other tissues. Normally malignant brain tumors contain cancer cells and are considered to be dangerous for infected persons because they spread rapidly into other parts of brain. There are more than 120 know varieties of tumors which belong to brain or nervous system of human body. The approaches pertaining to the diagnosis and treatment of these tumors are not unique. It is dependent upon the type of cells and the area of the existence of tumors inside human body. The diagnosis of brain tumor is a very complicated and challenging process due to the requirement of sophisticated medical equipments along with number of medical experts of different fields to ensure safety of the patient during the procedure. The early stage and timely detection of brain tumors is curable but the process of treatment is very costly and painful for the patient. Since brain is one of the most sophisticated as well as complex part of human organs which consists of more than a hundred billion nerves, so lot of precautions are involved while testing and treating brain tumors. Brain also provides the artificial intelligence support which controls our movement, thoughts, speech and also ensures the proper and smooth working of other parts of body. As an estimate 19 million new cancer cases along with 10 million deaths due to cancer are being reported per year across the globe. Most of these deaths are due late diagnosis of the cancer which reaches to last stage. Therefore the development of rapid, accurate, advance and cost effective mechanism for detection of malignant tumors is an essential requirement to make early stage and timely detection of tumors possible. In order to save the life of patient suffering from cancer the early stage and timely detection of tumor is the most necessary requirements^1,2^.

Presently available photonic bio-sensing technologies are amongst those which could address the challenges involved in the earlier stage and timely detection of malignancy in human body. In traditional bio-sensing techniques, bio-sensing devise has to be inserted inside the sample which is in the form of liquid called analyte on the other hand in photonic bio-sensing technology cavity region of the device has infiltrated by fluid sample under investigation^3–5^. Moreover such devices have several advantages like accuracy in results, cost effectiveness, rapid and fast processing of samples under investigation. Basically any biosensor based on photonic biosensing technology is consisted of five elementary parts. The first part is the fluid sample under investigation. It is known as analyte. The second part of the device is used for sensing the analyte under consideration. This part is identified as a bio-receptor. The information from bio-receptor is being sent to third part of the biosensor which is known as transducer. The working of transducer is to produce measurable signals. These measureable signals are being fetched in to the fourth part of biosensor which consists of an electronic circuit to produce digital signals. Finally the combination of hardware and software is used for display of results as an outcome of investigations^6–8^.

Photonic crystals (PhCs) are one of the most essential and integral part of photonic bio-sensing technology as discussed above^9–11^. Actually PCs are the multilayer thin film structures of periodically varying refractive index of the constituent materials. Depending upon the periodic modulation of refractive index of layers they can be classified into one-dimensional (1D), two-dimensional (2D) and three-dimensional (3D). PhCs have tremendous ability of controlling the propagation of electromagnetic waves (EMWS) passing through them due to the formation of photonic band gap (PBG). The remarkable PBG properties make them suitable to be used in designing the light driven modern photonic devices. The devices like optically driven photonic switches, reconfigurable photonic biosensors, PhCs based waveguides and filters are some examples of light driven modern devices which are playing a prominent role in the field of photonic engineering and technology^12,13^. Nowadays involvement of PhCs in the designing of biosensors for investigating various kinds of biomedical fluids has become new hot topic of research due to their simple design and easier fabrication^14–16^. For example high performance ultra-sensitive biosensor capable of detecting cancers cells proposed by Arafa et al.^17^ and Nouman et al. suggested a 1D PhC based biosensing design for rapid detection of brain lesions with ultra-high sensitivity of value 3080.80 nm/ RIU^18^.

Recently the development in the field of material engineering has provided us an opportunity to use of nanocomposite materials in the fabrication of photonic devices. Actually nanocomposite materials are those materials in which metal or dielectric nanoparticles are embedded into host materials. This inclusion of nanoparticles into host material results the relatively larger value of refractive index^19^ of the host material in contrast to the conventional dielectrics or metals. Such nanocomposite materials have found novel applications like fabrication of high quality optical lenses and microscope with high resolution capabilities. The size of the nanoparticle embedded into host material like metal, superconductor, dielectric and semiconductor may vary between 1 and 100 nm depending upon the requirement pertaining to the application^20^. The insertion of nanoparticles into host material reduces the absorption depending upon the volume fraction and radius of spherical nanoparticles. Moreover the technological development in the field of nanosciences attracts the attention of researchers to design and develop photonic biosensors made up of nanocomposite materials^21^. For example Pandey et al. presented how to use ZnO–TiO_2_ nanocomposite power pellets for morphological and relative humidity sensing applications^22^. Zhou et al., suggested a novel way of detecting breast cancer cells by using graphene oxide based nanocomposite^23^ materials.

From past few years researchers have given their attention for designing of photonic structures made up of dielectrics, semiconductors, metamaterials as well as metals^24–27^ of tremendous biosensing applications. Moreover application of superconductors (SCs) as a constituent material in the development of 1D PhCs has explored new possibility of making externally tunable photonic devices. The optical properties of such devices can easily be controlled by changing the temperature of ambient medium because refractive index of the superconductor depends upon London penetration depth which in turn depends upon temperature of surrounding environment. The break in periodicity of the constituent material layers of 1D PhCs by introducing a defect layer of SC results the existence of resonant mode of high transmission inside PBG of the defective PhCs. The position, intensity and full width half maximum (FWHM) of such resonant modes can easily be tuned with the help of changing the temperature of SC material externally^28^. This temperature dependent external tuning of resonant mode inside PBG has inspired the material scientists to develop the verity of nanocomposite materials with superconducting host. For example Ramanujam et al. suggested biosensing applications of 1D defective PhC in which defect layer of air is encapsulated between SiO_2_ embedded nanocomposite layers of Yttrium barium copper oxide^29^. Recently Malek et al. suggested 1 D defective PhC composed of nanocomposite superconducting material for detection of cancerous brain tumors^30^. Fatma et al. studied the identification of gamma-ray radiation using a 1D DPhC made up of polymer doped porous silicon photonic structures. They have infiltrated the pours of porous silicon 1D DPhC with crystal violet and carbon fuchsine. The periodicity of the structure has been broken by adding defect layer of poly-vinyl alcohol polymer at the middle of the structure^31^. Ayman et al. also investigated a pressure sensing hetero-structured ternary photonic crystal whose silicon dioxide and porous silicon layers have embedded quantum dots for amplify the sensitivity of the structure^32^. A novel approach for detecting tumor markers by using PhC doped with AlEgen has been suggested by Zhijun et al. They have used the method of infiltrating luminogens with aggregation-induced emission (AIEgens) for achieving higher sensitivity^33^. Diptimayee et al. suggested how sensing capabilities of PhC resonating structure with exponentially graded refractive index profile can be enhanced^34^. Zaky et al. suggested how 1D DPhCs can be utilized as an effective pressure sensor by using the mechanism of parity-time symmetry for monitoring the different kind of pressure variations of the gases emitted from the ground^35^.

The above mentioned piece of tremendous research work motivated us to contribute in the field of biomedical engineering by proposing a simple 1D defective photonic crystal (DPhC) based biosensor for detection of various malignant tissues of human brain because the early stage detection of malignant brain tissues is the one of essential requirement for prevention of the rapid growth of cancerous cells in human body. The proposed structure is capable of identifying the malignant tissues of human brain if any, by detecting the minute change in refractive index of sample containing brain tissues of various parts of human brain. The refractive index of sample containing different brain tissues varies due to change in the size of the tissues as well as their concentration. In the present work we have considered the variation of refractive index from 1.3333 to 1.4591. Apart from the detecting the malignancy in human brain our sensing design could also investigate other samples as well whose refractive index varies from 1.3333 to 1.4591. Thus our design can be considered as a refractive index sensor which can comfortably sense the refractive index variations from 1.3333 to 1.4591. The present research work deals with 1D DPhC whose air cavity is surrounded by SiO_2_ embedded nanocomposite high temperature superconductor buffer layers. To the best of our knowledge 1D DPhC composed of SiO_2_ nanocomposite buffer layers has been rarely used for investigating malignancy in human brain tissues.

## Biosensor design

**Figure 1:**
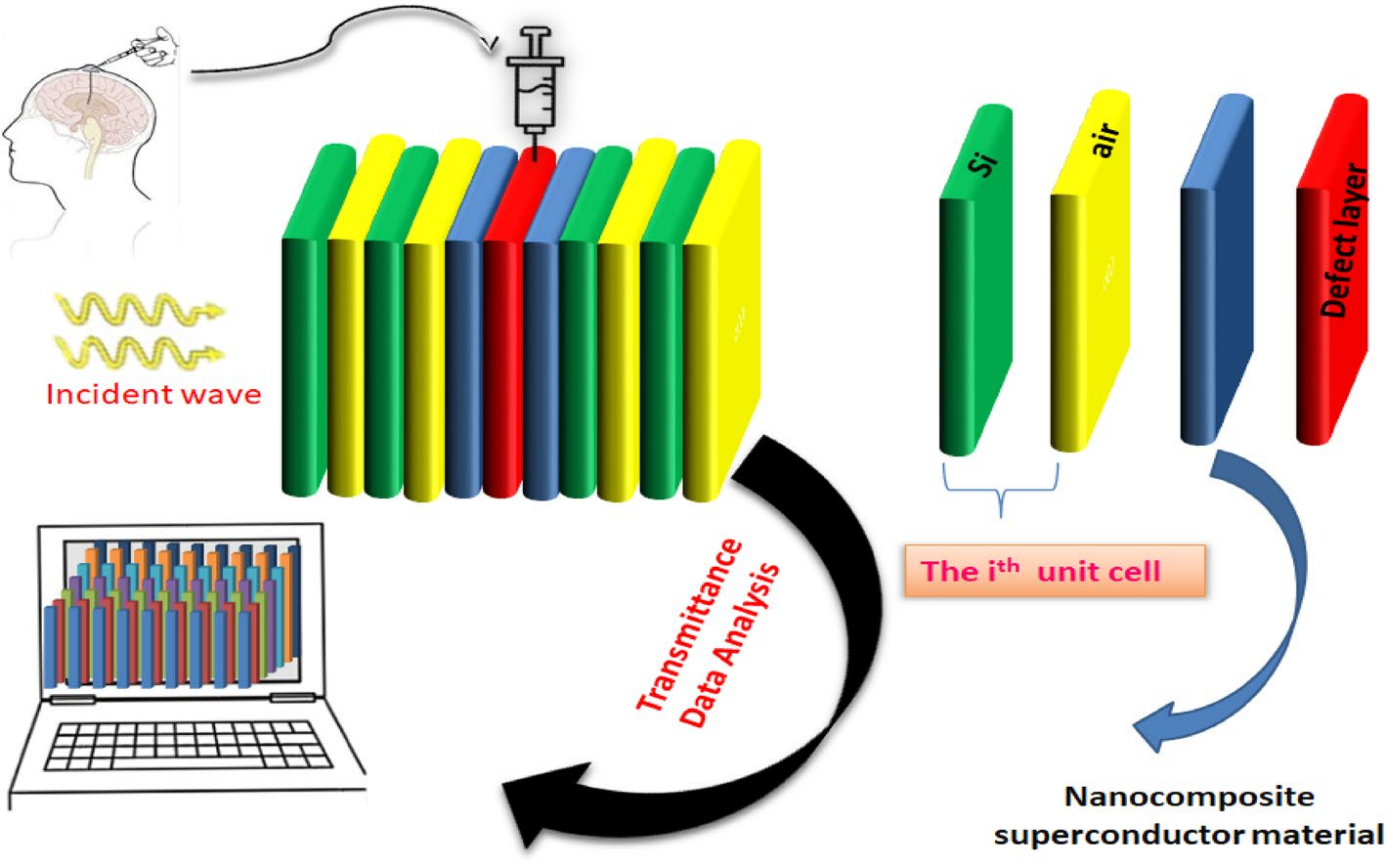
The schematics of proposed 1D photonic biosensor in which cavity region has been created by sandwiching pair of nanocomposite superconducting buffer layers either side of cavity region.

The proposed biosensing design (*AB*)^*N*^*CDC*(*AB*)^*N*^ is capable of sensing and detecting the malignancy in brain tissues. Here *A* and *B* are representing alternate layers of materials Si and air of two binary 1D PhCs (*AB*)^*N*^. The alphabet *N* has been used to represent the period number of each 1D PhC. The air cavity region *D* of the proposed structure has been surrounded either side by two buffer layers C of nanocomposite superconducting material^26,27^. The modified cavity region consisting of three layers of two materials is sandwiched between two identical 1D binary PhCs (*AB*)^N^ to form the proposed photonic biosensing design (*AB*)^N^*CDC*(*AB*)^N^ as depicted in Fig. 1. The lumber puncher procedure is applied to extract cerebrospinal fluid (CSF) samples containing different brain tissues with the help of long needle passes through human skull. After extraction the CSF sample is inserted into the air cavity region D of the proposed biosensor for examination as per the details shown in Fig. 1. Presently available thin film deposition technologies depending upon the material used in the design may be utilized to fabricate the proposed biosensing structure like spin coating, doctor-blade, dip coating approaches etc.

## Modeling and simulation

The refractive index (RI) and thickness of material layers A and B of 1D PhC (*AB*)^N^ used in the proposed design (*AB*)^N^*CDC*(*AB*)^N^ are being represented by *n*_1_, *n*_2_ and *d*_1_, *d*_2_ respectively. The refractive index and thickness of pair of SiO_2_ embedded nanocomposite superconductor material YBa_2_Cu_3_O_7_ are denoted by *n*_3_ and *d*_3_ respectively. The thickness of air cavity region *D* is d_4_. The various CSF samples under investigation will be poured into the cavity region one by one. The sum of the thicknesses of layers *A* and *B* is represented by *dd* = *d*_1_ + *d*_2_.

The refractive index of high temperature superconductor YBa_2_Cu_3_O_7_ is described with the help of the two-fluid model as^28–30^

1 $${n}_{sc}=\sqrt{1-\frac{{c}^{2}}{{\omega }^{2}{{\lambda }_{L}}^{2}}}$$

where *c*, *ω* and *λ*_L_ are representing speed of incident EMWs in free space, angular frequency of the incident EMWs and the temperature-dependent London penetration depth high temperature superconducting material YBa_2_Cu_3_O_7_, respectively. The expression of temperature-dependent London penetration depth is given as^21^

2 $${\lambda }_{L}\left(T\right)=\frac{{\lambda }_{o}}{\sqrt{1-{\left(\frac{T}{{T}_{c}}\right)}^{2}}}$$

here ${\lambda }_{o}$ is the London penetration depth at *T* = 0 K and *T*_*c*_ is the critical temperature of the superconducting material.

The effective permittivity (*ε*_*eff*_) of SiO_2_ embedded nanocomposite superconducting material is defined with the help of Maxwell–Garnett formula as^18,26^

3 $${\varepsilon }_{eff}= \frac{{\varepsilon }_{m} (2 \eta {\varepsilon }_{sc}-2 \eta {\varepsilon }_{m}+ {\varepsilon }_{sc}+ 2 {\varepsilon }_{sc})}{(2 {\varepsilon }_{m} +{\varepsilon }_{sc}+ \eta {\varepsilon }_{m}- \eta {\varepsilon }_{sc})}$$

where ${\varepsilon }_{m}$ is the permittivity of the dielectric material SiO_2_, ${\varepsilon }_{sc}$($= n_{sc}^{2}$) is the permittivity of high temperature superconductor material YBa_2_Cu_3_O_7_ and $\eta$ is representing the volume fraction of SiO_2_ material embedded into YBa_2_Cu_3_O_7_. The symbol *n*_*sc*_ is representing refractive index of YBa_2_Cu_3_O_7._

The transfer matrix formulation has been used to study the propagation of EMWs from air into the proposed 1D DPhC (AB)^N^CDC(AB)^N^ at an angle of incident *θ*_0_ as describe below^36–38^

4 $$M_{j} = \left( {\begin{array}{ll} {\cos \gamma _{j} } \hfill & \quad { - \frac{i}{{p_{j} }}\sin \gamma _{j} } \hfill \\ { - ip_{j} \sin \gamma _{j} } \hfill & \quad {\cos \gamma _{j} } \hfill \\ \end{array} } \right)$$

here the alphabet *j* has been used to represent transfer matrix of any layer* j* of the design. The value of *p*_j_ in *j*-th layer is $p_{j} = z_{0} n_{j} \cos \theta_{j}$ and $p_{j} = \frac{{\cos \theta_{j} }}{{z_{0} n_{j} }}$ for s and p polarized EMWs respectively. Here $\gamma_{j} = \frac{2\pi }{{\lambda_{0} }}n_{j} d_{j} \cos \theta_{j}$. The notations *d*_j_, *n*_j_ and *θ*_j_ are representing thickness, refractive index and ray angle of EMW inside *j*-th layer of the structure. The *z*_0_ and *λ*_0_ are used for representing free space impedance and free space wavelength respectively. In order to get total transfer matrix of whole structure we have extended the above formulation as

5 $$\begin{aligned} M_{T} & = \left( {X_{A} X_{B} } \right)^{N} X_{C} X_{D} X_{C} \left( {X_{A} X_{B} } \right)^{N} \\ & = \left( {\begin{array}{*{20}l} {M_{{11}} } \hfill & {M_{{12}} } \hfill \\ {M_{{21}} } \hfill & {M_{{22}} } \hfill \\ \end{array} } \right) \\ \end{aligned}$$

The elements of total transfer matrix *M*_T_ are represented by *M*_11_, *M*_12_, *M*_21_ and *M*_22_. The expression of transmission coefficient *t* of whole structure is given by

6 $$t = \frac{{2\eta_{0} }}{{(M_{11} + M_{12} \eta_{S} )\eta_{0} + (M_{21} + M_{22} \eta_{S} )}}$$

In Eq. (6) *η*_0_ and *η*_S_ have been used to represent an incident and exit media of the design. For *s* polarized EM wave $\eta_{0} = z_{0} n_{0} \cos \theta_{0} ,$ and $\eta_{S} = z_{0} n_{S} \cos \theta_{S}$ whereas for *p* polarized EM wave $\eta_{0} = \frac{{\cos \theta_{0} }}{{z_{0} n_{0} }}$ and $\eta_{S} = \frac{{\cos \theta_{S} }}{{z_{0} n_{S} }}.$

The transmittance (*T*) of the whole 1D DPhC (*AB*)^*N*^*CDC*(*AB*)^N^ is given by^36–38^

7 $$T = \frac{{\eta_{S} }}{{\eta_{0} }}\left| t \right|^{2}$$

## Results and discussions

In the present piece of research work we have studied the interaction between incident EMWs with the proposed biosensor composed of 1D DPhC. The transfer matrix method (TMM) and MATLAB software both have been used to carry out the simulations of entire research work. The refractive index and thickness of Si layer *A* is set to be *n*_*1*_ = 3.3 and *d*_*1*_ = 0.35 μm respectively. On the other hand the refractive index and thickness of air layer *B* has been chosen as *n*_*2*_ = 1and *d*_*2*_ = 0.275 μm respectively. The thickness *d*_*3*_ of both nanocomposite buffer layers is set to be 0.09 μm whose wavelength dependent dielectric constant has been obtained by using Eqs. (1)–(3). The thickness of cavity region has been chosen as *d*_*4*_ = *dd,* where *dd* = *d*_*1*_ + *d*_*2*_ is length of the period of 1D PhC (*AB*)^*N*^. The period number of both the 1D PhCs has been fixed to *N* = 2 to make our design as small as possible. The numeric value of London penetration depth (*λ*_*0*_) of high temperature superconductor (HTS) YBa_2_Cu_3_O_7_ at temperature 0 K has been taken to be 200 nm. The critical temperature of nanocomposite material layers has been set to 92 K. In this study we have fixed the ambient temperature (*T*) and volume fraction (*η*) of nanocomposite layers as 92 K and 0.2 respectively. The permittivity of dielectric material embedded into S*iO*_*2*_ has been taken to be 2.1025. We have considered air in place of substrate. In order to investigate the biosensing capabilities of proposed design we have plotted transmission spectra of the design with the help of Eqs. (4)–(7) and MATLAB software. All the simulations of the proposed design have chosen carried out at normal incidence i.e. *θ* = 0° in order to overcome the difficulties associated with oblique incidence. The results of proposed biosensing photonic structure can be obtained by connecting both the ends of proposed structure with single mode fiber (SMF). The use of SMF limits the wave vector criterion associated with the design during measurements. The white light source is connected to the one end of fiber which is joined with input end of the design. The other end of the fiber connected with output terminal of the proposed design is joined with optical spectrum analyzer (OSA) which projects the output results into monitor via computer.

The refractive index of various kinds of normal and abnormal brain cells are the most important parameters for investigating the biophotonic sensing capabilities of the proposed design. Such structures can be used in various kinds of biomedical applications for designing of biosensors to get economical, efficient and fast results. This research is focused towards the biosensing application of 1D DPhC for differentiating between various kind of normal and malignant brain lesions. Actually brain lesions are particular type of brain tumors which describe localized regions of the damaged brain tissues. The unaccepted growth of these tissues may cause deadly or malignant brain tumors which may affect the life of patient. The sample containing these lesions is collected by injecting thin and long needle through the brain or spinal cord of affected person with the help of lumbar puncture or spinal tap procedure respectively. This sample contains of colorless fluid around the human brain and spinal cord which ensure the needful supplies to the organs of human brain and spinal cord and extract the waste from those places. This fluid is known as Cerebrospinal fluid (CSF). The sample containing CSF becomes an essential requirement if the person has symptoms of autoimmune diseases, inflammation in spinal cord and/or brain tissues or leukemia due to severe kind of viral or bacterial infections in CSF as per the advice of medical practitioner. The CSF fluid of refractive index 1.3333 has been taken as an internal reference in contrast to the refractive index of pure distill-water solution of value 1.333 as an external reference in order to initiate the findings.

The degree of malignancy may be determined by the refractive index of various kinds of brain lesions present in the sample and is proportional to the refractive index of samples of various brain lesions. The threshold value of refractive index of brain lesions which helps in determining malignancy is 1.395 i.e. the brain lesion samples of refractive index less than this threshold value are non-malignant otherwise the sample is said to be malignant. Table 1 below gives the refractive index of various normal and damaged brain tissues which have been investigated in this research work. The variation in the RI of various brain tissues is due to the change in the value of protein level inside different brain tissues. The protein level in cancerous cells is very high which results large RI values^17,18^

**Table 1:** Different kinds of normal and damaged brain tissues along with their refractive indices^30^.

Classification	Brain tissues	Refractive index (RIU)
Normal	CSF	1.3333
	Wall of solid brain	1.3412
	Multi sclerosis	1.3425
	Oligodendroglioma	1.3531
	Gray matter	1.3951
Malignant	White matter	1.4121
	Low grade glioma	1.432
	Medulloblastoma	1.4412
	Glioblastoma	1.447
	Lymphoma	1.4591

**Figure 2:**
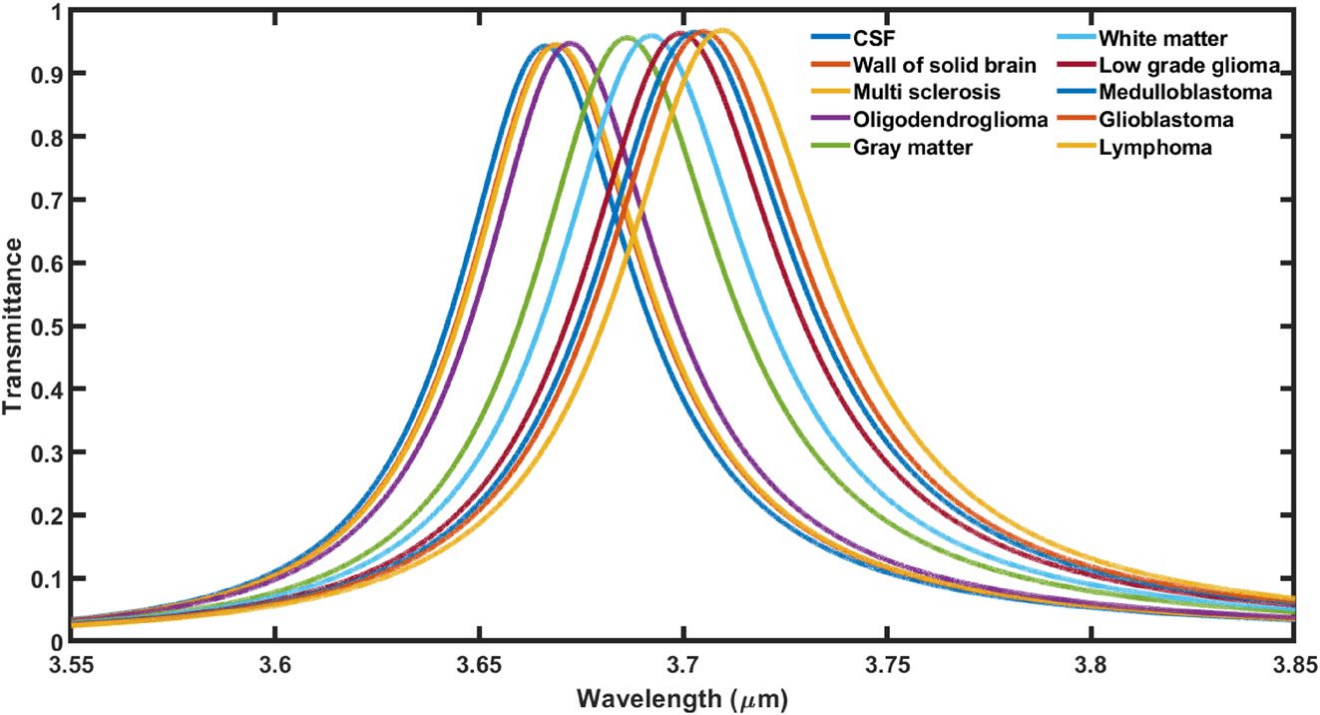
The transmittance spectra of 1D DPhC(AB)^2^CDC(AB)^2^ when cavity is loaded with ten different brain tissues in accordance with Table 1 and the thickness of cavity layer has been fixed to *d*_*4*_ = 0.625 µm.

First we pour fluid samples containing different brain tissues as per the information given in Table 1, into cavity region of proposed 1D DPhC (*AB*)^2^*CDC*(*AB*)^2^ to carryout investigations for identifying the malignancy of sample. All the investigations pertaining to the work are being done in infrared region of electromagnetic spectrum ranging from 3.5 to 3.8 µm. The transmission spectra as presented in Fig. 2 shows 10 closely spaced different resonant peaks corresponding to fluid samples containing different brain lesions as mentioned in Table 1 inside PBG of 1D DPhC (*AB*)^2^*CDC*(*AB*)^2^ which extends from 3.55 to 3.85 µm under normal incidence. The existence of the large PBG of width 0.30 µm is due to the high refractive index contrast between silicon and air layers of the proposed photonic structure. As the fluid sample under examination changes from CSF to Lymphoma (as per Table 1) one by one, the resonant peak inside PBG starts to move towards higher wavelength side as evident from Fig. 2. The shifting of resonant peaks inside PBG is observed with respect to the position of resonant peak corresponding to CSF sample. This shifting of resonant peaks inside PBG is in accordance with formulation of standing wave inside laser cavity as mentioned in Eq. (8). Actually the change in the refractive index of cavity region is due to presence of different lesions into the samples. This variation in the refractive index may alter the resonant condition governing the standing wave formulation inside cavity region. This alteration is responsible for the shifting of resonant peak towards higher wavelength side inside PBG in order to fix the optical path difference inside cavity region.

8 $$\Delta = m\lambda = n_{eff} G$$

here notations Δ, *m*, *λ*, *G* and *n*_*eff*_ have been used to represent optical path difference between standing waves inside cavity region, an integer, resonant wavelength inside cavity, effective refractive index of 1D DPhC (*AB*)^2^*CDC*(*AB*)^2^ and geometrical path difference respectively.

The validation of the proposed design as an efficient biosensor capable of detecting minute refractive index change is being done with the help of sensitivity calculation as per the formula given in Eq. (9). The sensitivity is one of the most popular parameters for evaluating the performance of the any biosensor as defined below in Eq. (9)^18,26–29^

9 $$S = \frac{\delta \lambda }{{\delta n}}$$

here δλ is the change in the position of central wavelength of resonant mode inside PBG due to corresponding change in refractive index (δn) of the samples under consideration. The CSF sample has been used as a reference to calculate *S* of the proposed structure when the cavity is loaded with different samples containing brain tissues seperately in accordance with Table 1. The numeric values of resonant wavelength (*λ*_*d*_) and senstivity (*S*) of the proposed design loaded with various samples seperately corresponding to cavity thickness *d*_4_ = *dd* are being given in Table 2 below.

**Table 2:** The performance evaluation table showing refractive index (RIU), resonant wavelength (*λ*_*d*_) and senstivity (*S*) of the proposed design when the cavity of thickness *dd* is loaded with different samples containing brain tissues.

Brain tissues	Refractive index (RIU)	λ_d_ (μm)	S (μm/RIU)
CSF	1.3333	3.6683	–
Wall of solid brain	1.3412	3.6694	0.13924
Multi sclerosis	1.3425	3.6711	0.30434
Oligod-endroglioma	1.3531	3.6745	0.31313
Gray matter	1.3951	3.6882	0.32200
White matter	1.4121	3.6953	0.34263
Low grade glioma	1.4320	3.7012	0.33333
Medulloblastoma	1.4412	3.7046	0.33642
Glioblastoma	1.4470	3.7074	0.34388
Lymphoma	1.4591	3.7114	0.34260

It has been observed from data of Table 2 that if cavity of the structure is loaded with different fluid samples containing brain tissues from CSF to lymphoma seperately, the senstivity of the proposed biosensor starts increasing from 0.13924 to 0.34260 µm/RIU corresponding to wall of solid brain and lymphoma tissues respectively. Thus the proposed structure possesses maximum senstivity value of 0.34260 µm/RIU corresponding to sample containing wall of solid brain tissues with cavity thickness *d*_*4*_ = 0.625 µm under normal incidence.

### The effect of increasing the thickness of cavity layer on the performance

**Figure 3:**
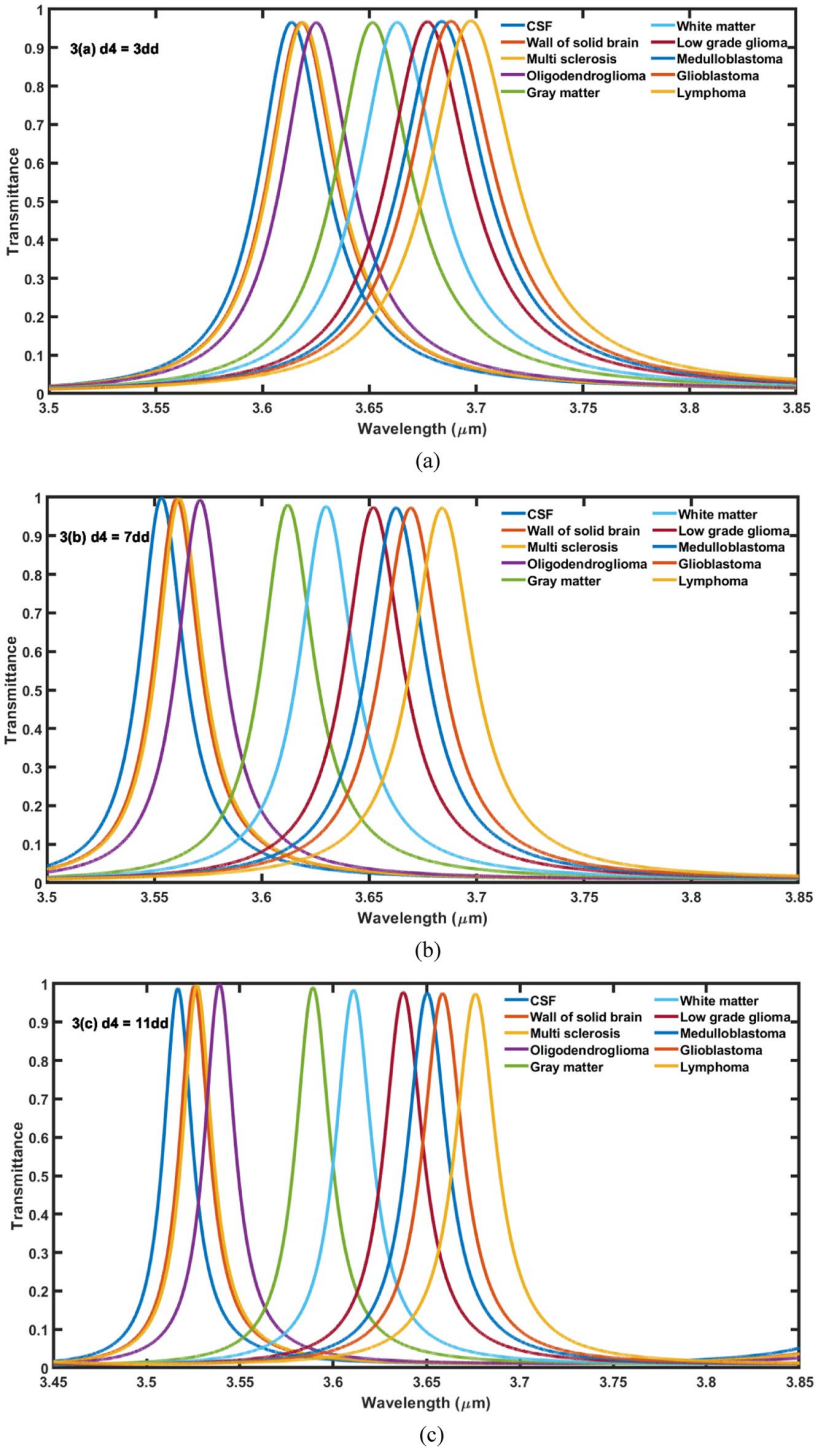

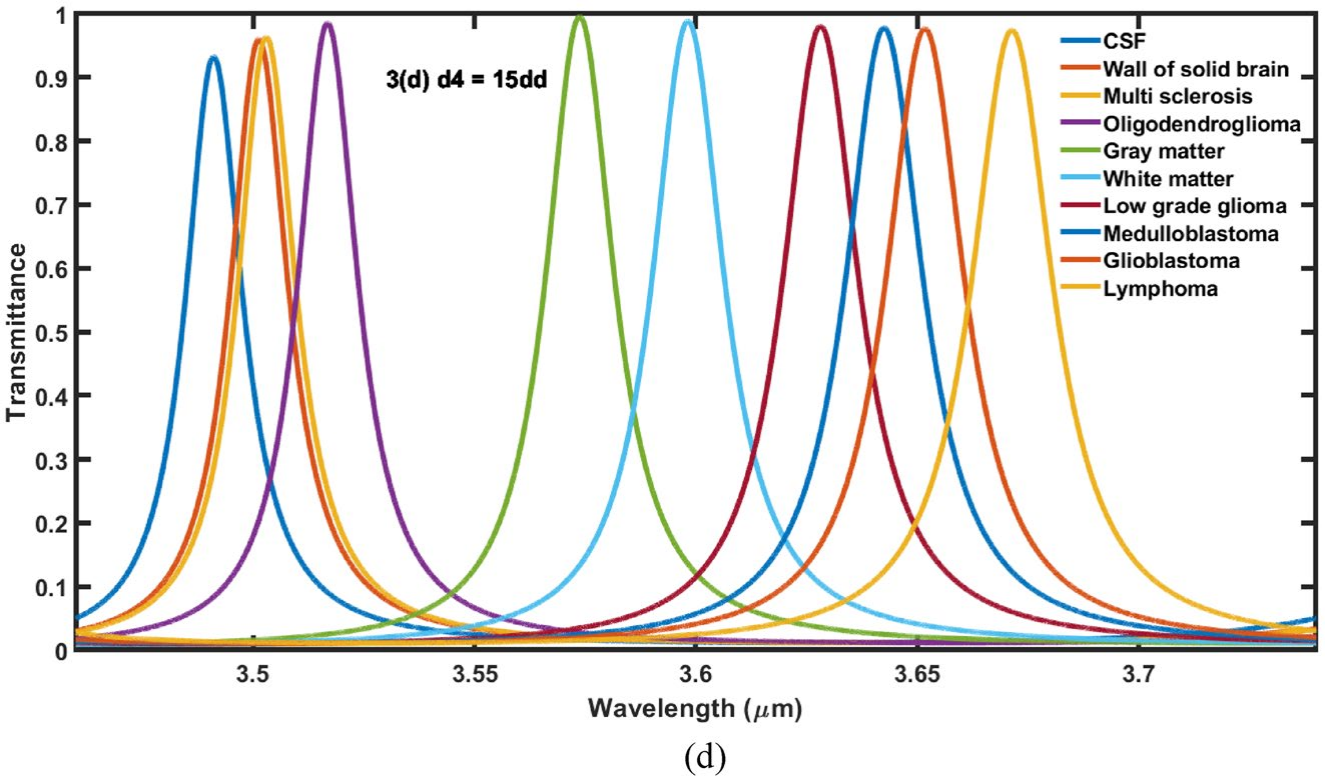
The transmittance spectra of 1D DPhC(*AB*)^2^*CDC*(*AB*)^2^ when cavity is loaded with ten different brain tissues in accordance with Table 1 under normal incidence. The thickness of cavity layer has been fixed to (**a**) *d*_*4*_ = 3*dd*, (**b**) *d*_*4*_ = 7*dd*, (**c**) *d*_*4*_ = 11*dd* and (**d**) *d*_*4*_ = 15*dd*.

In this section, we have studied the effect of changing the thickness of cavity region on the performance of the proposed 1D DPhC (*AB*)^2^*CDC*(*AB*)^2^under normal incidence. For this purpose the transmission spectra of proposed structure corresponding to different values of cavity layer thicknesses (*d*_*4*_) as 3*dd*, 7*dd*, 11*dd* and 15*dd* are being plotted in Fig. 3a–d, respectively. In this study we have fixed the values of *θ*_0_ = 0°, *η* = 0.2 and *T* = 4.5 K. Figure 3 shows that as thickness of cavity region increases from 3 to 15*dd* the central wavelength of resonant modes associated with the samples containing different brain tissues shifted to lower wavelength side.

**Figure 4:**
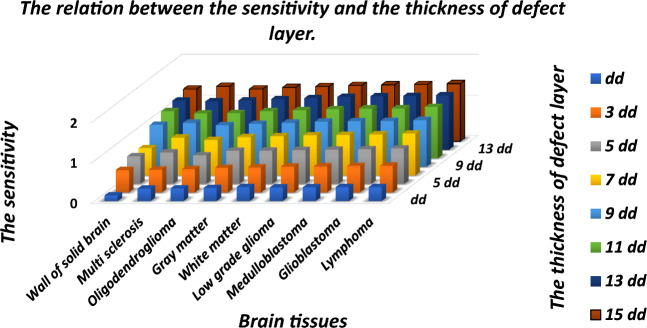
The 3D bar graph showing the sensitivity of the proposed 1D DPhC(*AB*)^2^*CDC*(*AB*)^2^ when cavity is loaded with ten different brain tissues in accordance with Table 1 at normal incidence corresponding to cavity layer thickness *d*_*4*_ which varies from *dd* to 15*dd* in steps of 2*dd*.

Moreover it has been also observed that the increase in the thickness of cavity layer also reduces the full width half maximum (FWHM) of each resonant mode inside PBG of proposed biosensor. The increase in the thickness of cavity layer from 3 to 7*dd* results significant improvement in the intensity of resonant peaks as evident from Fig. 3a,b, respectively. Further increase in the thickness reduces the intensity of transmission of resonant tunneling peaks inside PBG. There is one more interesting observation that the increase in the thickness of cavity layers from 7*dd* to 15*dd* in steps of 4*dd* results increase in the intensity of transmission of defect modes corresponding to samples containing gray matter and white matter respectively as evident in Fig. 3b–d. The intensity of resonant peaks corresponding to these two samples is reached to maximum corresponding to *d*_*4*_ = 15*dd*. The increase in the thickness of cavity layer increases the geometrical path of the ray of light inside cavity region which improves the interaction between light and fluid samples containing various brain tissues. This interaction results the variation in intensity of resonant peaks inside PBG of 1D DPhC (*AB*)^2^*CDC*(*AB*)^2^. The sensitivity of the structure corresponding to fluid sample containing lymphoma brain tissues reaches to *S* = 0.65898 μm/RIU, when the thickness of the cavity layer is increased from *dd* to 3*dd*. Further increase in the thickness of cavity layer results corresponding increase in sensitivity of the proposed design. It is reached to maximum of 1.42607 μm/RIU at *d*_4_ = 15*dd* when the cavity is loaded with sample having lymphoma tissues. Thus in order to design the photonic biosensors composed of 1D DPhCs, the appropriate thickness of cavity region can be one of the designing parameters to achieve optimum sensitivity value and hence performance. The sensitivity and central wavelength of resonant peak of the proposed design corresponding to different cavity layer thicknesses are being summarized in Tables 3 and 4 along with their pictorial representation in three dimensional bar graph as shown in Fig. 4 below, when cavity is loaded one by one separately with various types of brain tissue samples as per the data of Table 1.

**Table 3:** The performance evaluation table showing resonant wavelength (*λ*_*d*_) and senstivity (*S*) of the proposed design corresponding to different cavity layers of thicknesses *dd*, 3*dd*, 5*dd* and 7*dd* when cavity is loaded with ten different samples containing brain tissues.

Brain tissues	RI	*d*_*4*_ = *dd*		*d*_*4*_ = 3*dd*		*d*_*4*_ = 5*dd*		*d*_*4*_ = 7*dd*	
		λ_d_ (μm)	S (μm/RIU)	λ_d_ (μm)	S (μm/RIU)	λ_d_ (μm)	S (μm/RIU)	λ_d_ (μm)	S (μm/RIU)
CSF	1.3333	3.668	–	3.6158	–	3.5810	–	3.5553	–
Wall of solid brain	1.3412	3.669	0.13924	3.6202	0.55696	3.5864	0.68354	3.5623	0.66807
Multi-sclerosis	1.3425	3.671	0.30434	3.6210	0.56521	3.5882	0.78260	3.5639	0.93478
Oligod-endroglioma	1.3531	3.675	0.31313	3.6272	0.57575	3.5950	0.70707	3.5726	0.87373
Gray matter	1.3951	3.688	0.32200	3.6534	0.60841	3.6314	0.81553	3.6137	0.94498
White matter	1.4121	3.695	0.34263	3.6648	0.62182	3.6455	0.81852	3.6314	0.96573
Low grade glioma	1.4320	3.701	0.33333	3.6788	0.63829	3.6640	0.84093	3.6533	0.99290
Medulloblastoma	1.4412	3.705	0.33642	3.6855	0.64596	3.6728	0.85078	3.6638	1.00556
Glioblastoma	1.4470	3.707	0.34388	3.6899	0.65171	3.6787	0.85927	3.6706	1.01407
Lymphoma	1.4591	3.711	0.34260	3.6987	0.65898	3.6909	0.87360	3.6851	1.03179

**Table 4:** The performance evaluation table showing resonant wavelength (*λ*_*d*_) and senstivity (*S*) of the proposed design corresponding to different cavity thicknesses 9*dd*, 11*dd*, 13*dd* and 15*dd* when cavity is loaded with ten different samples containing brain tissues.

Brain tissues	RI	*d*_*4*_ = 9*dd*		*d*_*4*_ = 11*dd*		*d*_*4*_ = 13*dd*		*d*_*4*_ = 15*dd*	
		λ_d_ (μm)	S (μm/RIU)	λ_d_ (μm)	S (μm/RIU)	λ_d_ (μm)	S (μm/RIU)	λ_d_ (μm)	S (μm/RIU)
CSF	1.3333	3.5350	–	3.5185	–	3.5045	–	3.4927	–
Wall of solid brain	1.3412	3.5432	1.03797	3.5277	1.16455	3.5141	1.21518	3.5029	1.29113
Multi-sclerosis	1.3425	3.5450	1.08695	3.5287	1.10869	3.5155	1.19565	3.5052	1.35869
Oligod-endroglioma	1.3531	3.5553	1.02525	3.5407	1.12121	3.5286	1.21717	3.5183	1.29292
Gray matter	1.3951	3.6008	1.06472	3.5905	1.16504	3.5821	1.25566	3.575	1.33171
White matter	1.4121	3.6207	1.08756	3.6123	1.19035	3.6052	1.27791	3.5993	1.35279
Low grade glioma	1.4320	3.6452	1.11651	3.6387	1.21783	3.6335	1.30699	3.6291	1.38196
Medulloblastoma	1.4412	3.6569	1.12974	3.6515	1.23262	3.647	1.32066	3.6434	1.39666
Glioblastoma	1.4470	3.6645	1.13896	3.659	1.2357	3.6558	1.33069	3.6526	1.40633
Lymphoma	1.4591	3.6806	1.15739	3.678	1.26788	3.6746	1.35214	3.6721	1.42607

Figure 4 shows that the sensitivity of the proposed design under the influence of various samples of different brain tissues with respect to non-malignant sample containing CSF brain tissue. Figure 4 also shows that the increase in the thickness of cavity region also improves the sensitivity of the design due to improvement in the interaction between light and samples inside the cavity region. Besides this the sensitivity of the structure becomes stagnant if we further increase the thickness of cavity region. In this work we have focused our attention to improve the sensitivity of the structure by increasing the thickness of cavity region. On the same time we have also ensured the minimum size of the structure by limiting the period number of the 1D PhC (AB)^N^ to 2 which are fabricated either side of the cavity region containing buffer layers.

### The effect of increasing the volume fraction of nanocomposite SC buffer layers on the performance

**Figure 5:**
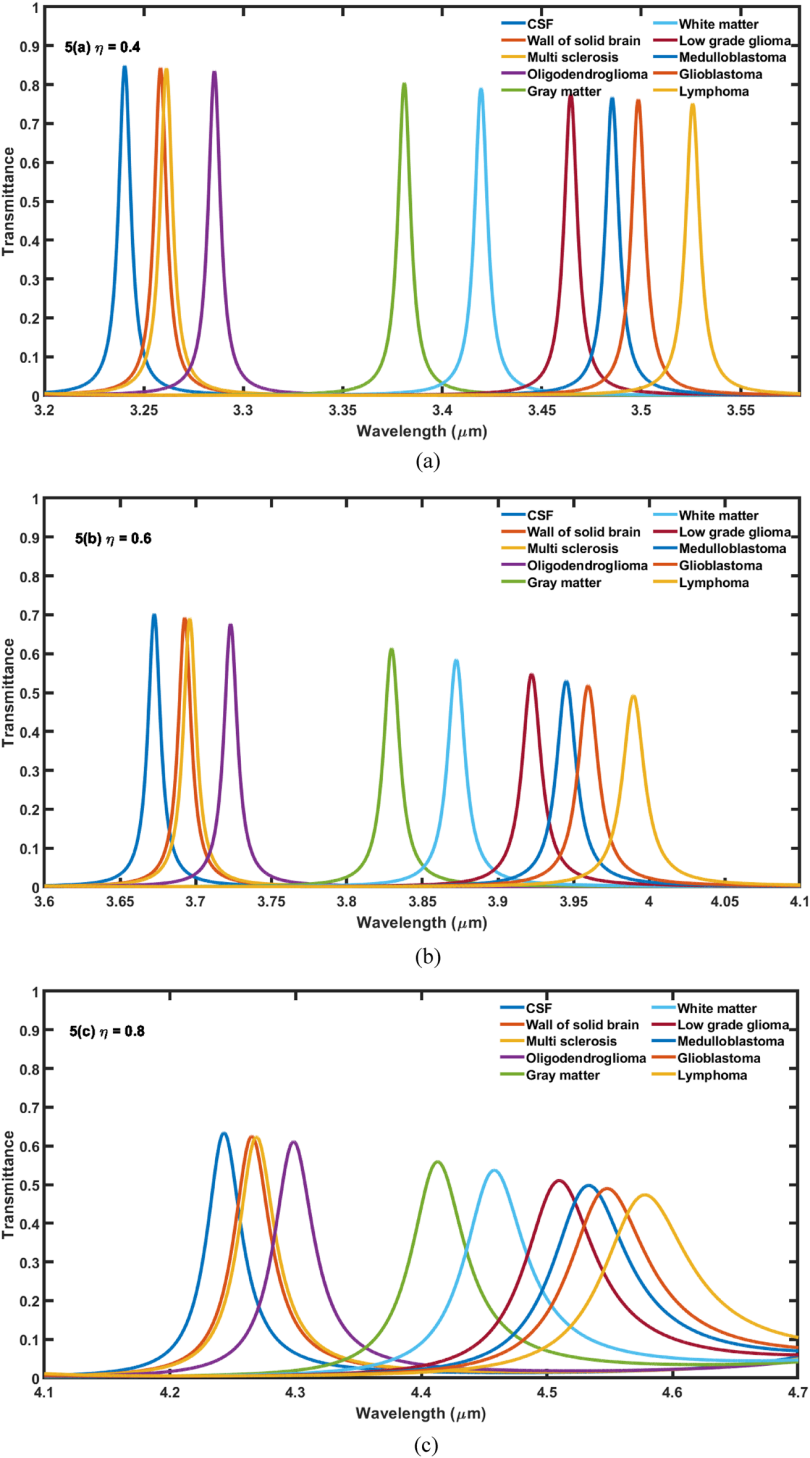
The transmittance spectra of 1D DPhC(*AB*)^2^*CDC*(AB)^2^ when cavity of thickness 15*dd* is loaded with ten different samples containing brain tissues in accordance with Table 1 at normal incidence. The volume fraction of nanocomposite pair of buffer layers fabricated either side of cavity layer is fixed to (**a**) *η* = 0.4, (**b**) *η* = 0.6 and (**c**) *η* = 0.8.

Next efforts have been made to study the effect of change in volume fraction *η* of nanocomposite superconducting buffer layers on the performance of the proposed 1D DPhC (*AB*)^2^*CDC*(*AB*)^2^ with cavity of thickness *d*_4_ = 15*dd* at *θ* = 0°. For this purpose the values of *η* have been changed from 0.2 to 0.8 in steps of 0.2. The transmittance of the proposed design corresponding to *η* = 0.4, 0.6 and 0.8 have been plotted in Fig. 5a–c respectively. Actually we wish to further increase the sensitivity of the design by changing *η* after optimizing thickness of cavity region as discussed in “**The effect of increasing the thickness of cavity layer on the performance**” Figure 5a below shows that the increase of *η* from 0.2 to 0.4 results the movement of resonant peaks inside PBG towards higher wavelength side in comparison to the previous results. Moreover this increase also improves FWHM of resonant peaks inside PBG corresponding to all samples. Additionally this change also results the gradual decrease in intensity of resonant peaks as we move lower side to higher side of PBG. This decrease in intensity is due to increase in refractive index of various brain tissue samples as described in Table 1. Further increase in the value of *η* from 0.4 to 0.8 in steps of 0.2 also decreases the intensity of the each resonant peaks corresponding to fluid samples containing wall of solid brain to lymphoma brain tissues in addition to the change in their central position and FWHM both.

**Figure 6:**
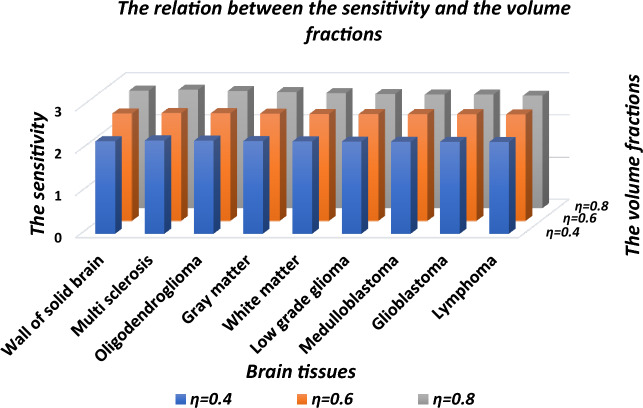
The 3D bar graph showing the sensitivity of the proposed 1D DPhC(*AB*)^2^*CDC*(*AB*)^2^ when cavity is loaded with ten different brain tissues in accordance with Table 1 at *d*_*4*_ = 15*dd* and *θ*_0_ = 0° corresponding to different values of volume fraction of nanocomposite SC buffer layers as 0.4, 0.6 and 0.8.

The visualization of the numeric data of Table 5 is being presented in Fig. 6. It depicts 3D bar graph showing sensitivity of the proposed design corresponding to three different values of volume fraction of nanocomposite superconducting buffer layers when cavity is loaded with various fluid samples containing different brain tissues as per the information given in Table 5. Figure 6 also shows that the increases in *η* from 0.4 to 0.8 in steps of 0.2 also increases the sensitivity of the design loaded with each sample of different brain tissues. The sensitivity attains to maximum of 2.80434 µm/RIU with *η* = 0.8 corresponding to multi sclerosis brain tissue sample as evident in Table 5. At fixed value of *η* the sensitivity is almost same for all samples under investigation as evident in Fig. 6.

**Table 5:** The performance evaluation table showing resonant wavelength (*λ*_*d*_) and senstivity (*S*) of the proposed design with *d*_4_ = 15*dd* and *θ* = 0° corresponding to nanocomposite SC buffer layers of different values of volume fraction (*η*) as 0.4, 0.6 and 0.8 when cavity is loaded with ten different samles containing brain tissues.

Brain tissues	RI	*η* = 0.4		*η* = 0.6		*η* = 0.8	
		*λ*_d_ (μm)	*S* (μm/RIU)	*λ*_d_ (μm)	*S* (μm/RIU)	*λ*_d_ (μm)	*S* (μm/RIU)
CSF	1.3333	3.2309	–	3.6728	–	4.2431	–
Wall of solid brain	1.3412	3.2482	2.18987	3.6929	2.5443	4.2651	2.78481
Multi sclerosis	1.3425	3.2512	2.20652	3.6963	2.55434	4.2689	2.80434
Oligodendroglioma	1.3531	3.2745	2.20202	3.7233	2.5505	4.298	2.77272
Gray matter	1.3951	3.3664	2.19255	3.8298	2.54045	4.4128	2.74595
White matter	1.4121	3.4033	2.18781	3.8724	2.53299	4.4579	2.72588
Low grade glioma	1.432	3.4463	2.18237	3.9226	2.5309	4.5099	2.70314
Medulloblastoma	1.4412	3.4662	2.18072	3.9454	2.52641	4.5331	2.68767
Glioblastoma	1.447	3.4786	2.17854	3.9599	2.52506	4.5487	2.68777
Lymphoma	1.4591	3.5045	2.17488	3.9898	2.51987	4.5779	2.66136

The sensitivity of the proposed 1D DPhC (*AB*)^2^*CDC*(*AB*)^2^ can be further increased to maximum by increasing the volume fraction of nanocomposite superconducting buffer layers of the cavity apart from the optimizing the thickness of cavity region. In this study we have explored the idea to improve the sensitivity of design by optimizing the two internal parameters associated with the cavity as *d*_4_ and *η* instead of optimizing the external parameters of the structure as temperature of superconducting nanocomposite layer as well as angle of incidence.

Finally, we have compared the performance of the proposed work with the similar kind of biosesning work based on photonic and plasmonic structutres for establishing the superiority of our work. Table 6 highlights the major features which are important for comparing the performance of any biosesning designs based on photonic structures at large. The comparison shows that the sensitivity of our design reacheses to maximum of 2804.34 nm/RIU (2.80434 µm/RIU) when the structure is loaded with sample containing multi sclerosis brain tissues as seen from Table 5.

**Table 6:** Comparison between the performance of proposed design with other biosensing designs of similar nature.

Structure	Topology	Refractive index range of various analytes	Sensitivity (nm/RIU)	FOM (Per RIU)	Q-factor	References	Year
SOI based 1D PhC	PBG	Sucrose (1.345–1.442)	1016.35	N.C	N.C	^39^	2021
1D PhC	PBG	Human Urine (1.335–1.347)	1360.02	N.C	N.C	^40^	2021
1D PhC	PBG and Tamm Resonance	Gas sensing (1.00–1.010)	1588.983	355.384	N.C	^41^	2021
1D PhC	PBG and Plasmonics	Cancer cell (1.36–1.38)	714.3	60.1	80.16	^42^	2022
2D PhC	PBG	Red blood cells (1.336–1.399)	898.9764	N.C	212.771	^43^	2022
Our work (1D PhC)	**PBG**	**Cancer cell (1.3333–1.4591)**	**2804.34**	**N.C**	**N.C**	**–**	**–**

(N.C. means not calculated).

Significant values are in [bold].

## Conclusion

The aim of the proposed work is to design an efficient and sensitive 1D photonic biosensor capable of detecting malignancy in different samples of brain tissues. The cavity of proposed the 1D DPhC (*AB*)^2^*CDC*(*AB*)^2^ is composed of pair of nanocomposite SC buffer layers fabricated either side of air cavity region to improve the interaction of light and fluid sample under investigation. The TMM and MATLAB software both have been used to carry out the simulation pertaining to the work. The performance of the proposed design has been evaluated in terms of sensitivity of the proposed design loaded with various brain tissue samples. The sensitivity of the proposed design has been improved by increasing the thickness of cavity layer in addition to volume fraction of nanocomposite SC buffer layers. We have obtained the optimized values of cavity layer thickness as well as volume fraction of nanocomposite superconducting buffer layers as 15dd and 0.8 respectively to get maximum sensitivity from the design. The sensitivity of the proposed design loaded with lymphoma sample of cavity layer thickness 15*dd* is 1.42607 μm/RIU. This value of the sensitivity can easily be reached to *S* = 2.66136 μm/RIU by changing *η* from 0.4 to 0.8 when the cavity is loaded with multi sclerosis brain tissue samples. The findings of this study are very useful for designing various biosensing structures which have significant and decisive role in the field of biomedical applications.

## Data Availability

The datasets used and/or analyzed during the current study are available from the corresponding author on reasonable request.

## References

[1] World Health Organization. Report on the health of refugees and migrants in the WHO European region: no public health without refugee and migrant health. (2018).

[2] da Silva, A. R. C. et al Application for predicting breast cancer through Google Prediction API. Anais da VI Escola Regional de Computaçãoaplicada à Saúde. SBC (2018).

[3] Shalaby AS, Alamri S, Mohamed D, Aly AH, Awasthi SK, Matar ZS, Tammam MT (2021). Theoretical study of one-dimensional defect photonic crystal as a high-performance sensor for water-borne bacteria. Opt. Quant. Electron..

[4] Aly AH, Awasthi SK, Mohamed AM, Al-Dossari M, Matar ZS, Mohaseb MA, Amin AF (2021). 1D reconfigurable bistable photonic device composed of phase change material for detection of reproductive female hormones. Phys. Scr..

[5] Aly AH, Awasthi SK, Mohamed AM, Matar ZS, Mohaseb MA, Al-Dossari M, Sabra W (2021). Detection of reproductive hormones in females by using 1D photonic crystal-based simple reconfigurable biosensing design. Crystals.

[6] Aly AH, Mohamed D, Mohaseb MA, Abd El-Gawaad NS, Trabelsi Y (2020). Biophotonic sensor for the detection of creatinine concentration in blood serum based on 1D photonic crystal. RSC Adv..

[7] Gandhi S, Awasthi SK, Aly AH (2021). Biophotonic sensor design using a 1D defective annular photonic crystal for the detection of creatinine concentration in blood serum. RSC Adv..

[8] Abd El-Ghany SE, Nouman WM, Matar ZS, Zaky ZA, Aly AH (2020). Optimized bio-photonic sensor using 1D-photonic crystals as a blood hemoglobin sensor. Phys. Scr..

[9] Vijayalakshmi D, Manimegalai CT, Ayyanar N, Vigneswaran D, Kalimuthu K (2021). Detection of blood glucose with hemoglobin content using compact photonic crystal fiber. IEEE Trans. Nanobiosci..

[10] Patel SK, Parmar J, Sorathiya V, Nguyen TK, Dhasarathan V (2021). Tunable infrared metamaterial-based biosensor for detection of hemoglobin and urine using phase change material. Sci. Rep..

[11] Aly AH, Awasthi SK, Mohamed D, Matar ZS, Al-Dossari M, Amin AF (2021). Study on a one-dimensional defective photonic crystal suitable for organic compound sensing applications. RSC Adv..

[12] Awasthi SK, Malaviya U, Ojha SP (2006). Enhancement of omnidirectional total-reflection wavelength range by using one-dimensional ternary photonic bandgap material. JOSA B.

[13] Srivastava R, Pati S, Ojha S (2008). Enhancement of omnidirectional reflection in photonic crystal heterostructures. Progress Electromagnet. Res. B.

[14] Aly, A. H., Mohamed, D., Zaky, Z. A., Matar, Z. S., Abd El-Gawaad, N. S., Shalaby, A. S., &Mohaseb, M. Novel biosensor detection of tuberculosis based on photonic band gap materials. *Mater. Res*. **24**, (2021).

[15] Hassan S, Elshahat K, Eissa M (2021). Dosimetric evaluation of physical parameters for different energies in advance radiotherapy technique for Liver cancer. Arab. J. Nucl. Sci. Appl..

[16] Aly AH, Nouman WM, Abd El-Ghany SES, Sallam SM, Dawood AFB (2019). Theoretical studies on hemoglobin periodic structure sensor. Exp. Theo. Nanotechnol..

[17] Aly AH, Zaky ZA (2019). Ultra-sensitive photonic crystal cancer cells sensor with a high-quality factor. Cryogenics.

[18] Nouman WM, El-Ghany A, Sallam SM, Dawood AFB, Aly AH (2020). Biophotonic sensor for rapid detection of brain lesions using 1D photonic crystal. Opt. Quant. Electron..

[19] Shi C, Owusu KA, Xu X, Zhu T, Zhang G, Yang W, Mai L (2019). 1D carbon-based nanocomposites for electrochemical energy stroge. Small.

[20] Ramanujam NR, Wilson KJ (2016). Optical properties of silver nanocomposites and photonic band gap–pressure dependence. Opt. Commun..

[21] Elsayed HA (2018). Transmittance properties of one-dimensional ternary nanocomposite photonic crystals. Mater. Res. Express.

[22] Pandey NK, Tiwari K, Roy A (2012). ZnO–TiO2 nanocomposite: Characterization and moisture sensing studies. Bull. Mater. Sci..

[23] Zhou J, Zheng Y, Liu J, Bing X, Hua J, Zhang H (2016). A paper-based detection method of cancer cells using the photo-thermal effect of nanocomposite. J. Pharm. Biomed. Anal..

[24] Aly AH, Ryu SW, Hsu HT, Wu CJ (2009). THz transmittance in one-dimensional superconducting nanomaterial-dielectric superlattice. Mater. Chem. Phys..

[25] Aly AH, Mohamed D (2015). BSCCO/SrTiO3 one dimensional superconducting photonic crystal for many applications. J. Supercond. Novel Magn..

[26] Vetrov SY, Avdeeva AY, Timofeev IV (2011). Spectral properties of a one-dimensional photonic crystal with a resonant defect nanocomposite layer. J. Expe. Theor. Phys..

[27] Moiseev SG (2011). Thin-film polarizer made of heterogeneous medium with uniformly oriented silver nanoparticles. Appl. Phys. A.

[28] Upadhyay M, Awasthi SK, Shiveshwari L, Shukla SN, Ojha SP (2015). Two channel thermally tunable band-stop filter for wavelength selective switching applications by using 1D ternary superconductor photonic crystal. J. Supercond. Novel Magn..

[29] Ramanujam NR, El-Khozondar HJ, Dhasarathan V, Taya SA, Aly AH (2019). Design of one dimensional defect based photonic crystal by composited superconducting material for bio sensing applications. Physica B.

[30] Malek C, Al-Dossari M, Awasthi SK, Matar ZS, Abd El-Gawaad NS, Sabra W, Aly AH (2022). Employing the defective photonic crystal composed of nanocomposite superconducting material in detection of cancerous brain tumors biosensor: Computational study. Crystals.

[31] Sayed FA, Elsayed HA, Mehaney A, Eissa MF, Aly AH (2023). A doped-polymer based porous silicon photonic crystal sensor for the detection of gamma-ray radiation. RSC Adv..

[32] Ameen AA, Al-Dossari M, Zaky ZA, Aly AH (2023). Studying the effect of quantum dots and parity-time symmetry on the magnification of topological edge state peak as a pressure sensor. Synth. Met..

[33] Liao Z, Zhou Q, Gao B (2023). AIEgens-doped photonic crystals for high sensitivity fluorescence detection of tumor markers. Biosensors.

[34] Dash D, Saini J, Goyal AK, Massoud Y (2023). Exponentially index modulated nanophotonic resonator for high-performance sensing applications. Sci. Rep..

[35] Zaky ZA, Al-Dossari M, Sharma A, Aly AH (2023). Effective pressure sensor using the parity-time symmetric photonic crystal. Phys. Scr..

[36] Born, M. Emil Wolf Principles of Optics. Electromagnetic Theory of Propagation, Interference (1980).

[37] Orfanidis, S.J. et al Multilayer film applications. Electromagnetic Waves and Antennas, 227–250. http://eceweb1.rutgers.edu/~orfanidi/ewa/

[38] Yeh P, Hendry M (1990). Optical waves in layered media. Phys. Today.

[39] Panda A, Pukhrambam PD, Keiser G (2021). Realization of sucrose sensor using 1D photonic crystal structure vis-à-vis band gap analysis. Microsyst. Technol..

[40] Jalil AT, Ashfaq S, Bokov DO, Alanazi AM, Hachem K, Suksatan W, Sillanpää M (2021). High-sensitivity biosensor based on glass resonance PhCcavities for detection of blood component and glucose concentration in human urine. Coatings.

[41] Zaky ZA, Aly AH (2021). Gyroidal graphene/porous silicon array for exciting optical Tamm state as optical sensor. Sci. Rep..

[42] Khani S, Hayati M (2022). Optical biosensors using plasmonic and photonic crystal band-gap structures for the detection of basal cell cancer. Sci. Rep..

[43] Rashidnia A, Pakarzadeh H, Hatami M, Ayyanar N (2022). Photonic crystal-based biosensor for detection of human red blood cells parasitized by plasmodium falciparum. Opt. Quant. Electron..

